# LOPAC library screening identifies suramin as a TRIM21 binder with a unique binding mode revealed by crystal structure

**DOI:** 10.1107/S2053230X25000913

**Published:** 2025-02-16

**Authors:** Yeojin Kim, Stefan Knapp, Andreas Krämer

**Affiliations:** a Department of Pharmacy Goethe University Frankfurt Max-von-Laue Strasse 9 Frankfurt am Main 60438Hessen Germany; University of São Paulo, Brazil

**Keywords:** TRIM21, E3 ligases, crystal structure, suramin

## Abstract

Differential scanning fluorimetry screening of the Library of Pharmacologically Active Compounds (LOPAC) identified four hits for the PRYSPRY domain of the human E3 ligase tripartite motif-containing protein 21 (TRIM21). Isothermal titration calorimetry subsequently confirmed suramin as a binder with micromolar affinity. To further investigate the binding mechanism, mouse TRIM21 was used as a structural surrogate due to its improved protein stability and high sequence similarity to the human counterpart. A crystal structure of the complex refined at 1.3 Å resolution revealed a unique binding mode, providing new avenues for targeting TRIM21 and for the development of proteolysis-targeting chimeras (PROTACs).

## Abstract 2

This communication describes the crystal structure of TRIM21 in complex with suramin, providing new opportunities to target this E3 ligase.

## 1. Introduction

**Figure 1:**
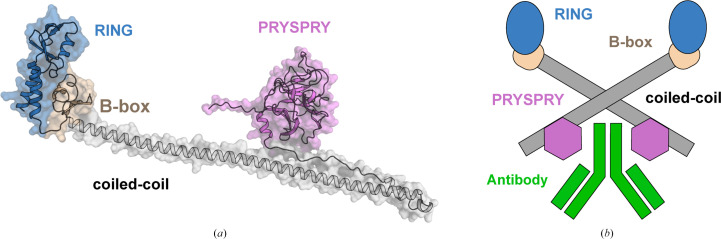
(*a*) Full-length human TRIM21 structure predicted by the *AlphaFold* server (Jumper *et al.*, 2021 ▸). The different domains are indicated by colour. (*b*) Scheme of the proposed dimerization and antibody binding. The scheme was adapted from Kiss & James (2022 ▸). The domains are coloured as in (*a*) and the antibody is shown in green.

Tripartite motif-containing protein 21 (TRIM21) is part of the TRIM family of E3 ligases and consists of a really interesting new gene (RING) finger domain, a B-box motif, a coiled-coil region and a PRYSPRY domain, named after the sp1A/ ryanodine receptor (Fig. 1 ▸*a*). TRIM21 plays a crucial role in immune defence, acting as an E3 ubiquitin ligase that facilitates the ubiquitination and degradation of target proteins via the proteasome pathway (James *et al.*, 2007 ▸). TRIM21 has a unique position within the TRIM family due to its ability to specifically bind to the Fc region of intracellular antibodies, resulting in the degradation of antibody-coated proteins or of non-enveloped viral particles (Dickson *et al.*, 2018 ▸; Keeble *et al.*, 2008 ▸; Fig. 1 ▸*b*). This particular function of TRIM21 has been exploited in the development of the TRIM-Away technology, which enables the selective and relatively rapid depletion of endogenous proteins in cells. However, a limitation of this method is the requirement for the exogenous delivery of specific antibodies into cells via microinjection or electroporation, rendering it impractical for most therapeutic applications (Clift *et al.*, 2017 ▸, 2018 ▸; Mevissen *et al.*, 2023 ▸). Recently, another comprehensive study was published by Lu and coworkers presenting heterobifunctional degraders based on acepromazine as a TRIM21 ligand (Lu *et al.*, 2024 ▸). However, the role of acepromazine as a sedative and neuroleptic drug and its modest activity towards human wild-type TRIM21 leave room for significant improvement. Nevertheless, these studies clearly demonstrated that TRIM21 can effectively be hijacked as an E3 ligase for targeted protein degradation (TPD).

Suramin was developed by Bayer more than 100 years ago for the treatment of African sleeping sickness. Structurally, suramin is a polysulfonated naphthylurea, with a comparably high molecular weight (1297 g mol^−1^) for a small molecule (Lipinski *et al.*, 2001 ▸) and carrying negative charges based on its sulfate groups, allowing it to interact with a broad range of biological targets (Steverding & Troeberg, 2023 ▸). Suramin has been studied as a treatment for several diseases and has been shown to bind to diverse proteins, including the spike protein of SARS-CoV-2 (Kwon *et al.*, 2023 ▸), alpha-thrombin (Lima *et al.*, 2009 ▸), *Leishmania mexicana* pyruvate kinase (Morgan *et al.*, 2011 ▸), human sirtuin homolog 5 (Schuetz *et al.*, 2007 ▸) and Ebola virus polymerase complex (Yuan *et al.*, 2022 ▸). Recently, it has also been shown to bind and inhibit the cullin–RING E3 ubiquitin ligase by disrupting its ability to recruit the E2 enzyme Cdc34 (Wu *et al.*, 2016 ▸). Despite its therapeutic potential, suramin is limited by its poor cell permeability, the need for intravenous administration and its toxicity (Steverding & Troeberg, 2023 ▸). Its binding to diverse proteins further contributes to off-target effects, which limit its broader clinical use.

In this study, we performed a screening campaign using a thermal shift assay to identify initial ligands for the PRYSPRY domain of TRIM21. Screening of the Library of Pharmacologically Active Compounds (LOPAC) yielded four potential hits. However, isothermal titration calorimetry (ITC) subsequently confirmed suramin as the only verified binder. To understand its mode of binding, we solved the high-resolution crystal structure of the murine TRIM21 PRYSPRY domain in complex with suramin at 1.3 Å resolution.

## 2. Materials and methodss

### 2.1. Expression and purification

The human TRIM21 PRYSPRY domain (residues Val287–Leu465; UniProt ID P19474) with an N-terminal His_6_-tag followed by a SUMO tag in a pET His_6_ SUMO LIC vector (Addgene) was transformed into *Escherichia coli* BL21(DE3) cells. The bacteria were grown in 4 l Terrific Broth medium containing 50 mg ml^−1^ kanamycin at 310 K. Protein expression was induced at an OD_600_ of 1.7 using 0.5 m*M* isopropyl β-d-1-thiogalactopyranoside (IPTG) at 291 K for 12 h. The cells were lysed by sonication in lysis buffer consisting of 50 m*M* MOPS pH 6.9, 800 m*M* NaCl, 10 m*M* imidazole, 1 m*M* TCEP. After centrifugation, the supernatant was loaded onto a 5 ml Ni Sepharose column equilibrated with 30 ml lysis buffer. The column was washed with 60 ml lysis buffer. Proteins were eluted using an imidazole step gradient (50, 100, 200 and 300 m*M*). Fractions containing the protein were pooled and the tag was cleaved by adding His-tagged SUMO protease (Sigma–Aldrich, catalogue No. SAE0067; protein:protease ratio ∼20:1) and dialyzing overnight at 277 K against size-exclusion (SEC) buffer (25 m*M* MOPS pH 6.9, 250 m*M* NaCl, 0.5 m*M* TCEP). The cutoff of the membrane was 3.5 kDa. The next day, a second Ni Sepharose column (2 ml) was used to remove uncleaved protein and the tag. The flowthrough was collected, concentrated to approximately 5 ml and loaded onto a Superdex 75 16/600 HiLoad gel-filtration column equilibrated with SEC buffer. The purity of the protein was assessed by SDS–PAGE and pure protein fractions were concentrated to approximately 10 mg ml^−1^ (determined by spectral absorbance with a NanoDrop). The yield per litre of medium was about 1–2 mg.

Mouse TRIM21 PRYSPRY domain (residues Val291–Met470; UniProt ID Q62191) with an N-terminal His_6_-tag was transformed into *E. coli* BL21(DE3) cells. The bacteria were grown in 4 l Terriﬁc Broth medium containing 100 mg ml^−1^ ampicillin at 310 K. Protein expression was induced at an OD_600_ of 1.7 using 0.5 m*M* IPTG at 291 K for 12 h. The cells were lysed by sonication in lysis buffer consisting of 50 m*M* Tris–HCl pH 7.8, 500 m*M* NaCl, 5% glycerol, 10 m*M* imidazole, 1 m*M* TCEP. After centrifugation, the supernatant was loaded onto a 5 ml Ni Sepharose column equilibrated with 30 ml lysis buffer. The column was washed with 60 ml lysis buffer. Proteins were eluted using an imidazole step gradient (50, 100, 200 and 300 m*M*). Fractions containing protein were pooled together and loaded onto a Superdex 75 16/600 HiLoad gel-ﬁltration column equilibrated with SEC buffer (25 m*M* Tris–HCl pH 7.8, 150 m*M* NaCl, 0.5 m*M* TCEP). The purity of the protein was assessed by SDS–PAGE and pure protein fractions were concentrated to approximately 10 mg ml^−1^. The yield per litre of medium was about 3–4 mg. Macromolecule-production information is summarized in Table 1 ▸.

### 2.2. Crystallization

Purified mouse TRIM21 PRYSPRY domain at 10 mg ml^−1^ in SEC buffer was mixed with suramin (50 m*M* stock solution in DMSO) to a final concentration of approximately 1 m*M* (final DMSO concentration of 2%). This protein–ligand complex was co-crystallized using the sitting-drop vapour-diffusion method in a 1:1 ratio with a reservoir solution consisting of 20% PEG 6000, 0.2 *M* ammonium chloride, 10% ethylene glycol. Rod-shaped crystals of TRIM21 grew to full size (∼10 × 50 µm) within 3–5 days and were subsequently determined to belong to space group *P*2_1_2_1_2_1_ with a single monomer in the asymmetric unit. Before flash-cooling the crystals, the ethylene glycol concentration was increased to 20% for cryoprotection. Details are summarized in Table 2 ▸.

### 2.3. Data collection

Diffraction data were collected on the I03 beamline at Diamond Light Source, Didcot, UK at a wavelength of 0.97625 Å at 100 K. Data were automatically processed using *autoPROC* (Vonrhein *et al.*, 2011 ▸) and scaled with *AIMLESS* (Evans & Murshudov, 2013 ▸). The mouse TRIM21 PRYSPRY-domain structure (PDB entry 2vok; Keeble *et al.*, 2008 ▸) was used as an initial search model for molecular replacement using *MOLREP* (Lebedev *et al.*, 2008 ▸). The dictionary file for the ligand already existed in the PDB under the three-letter code SVR. The final model was built manually using *Coot* (Emsley & Cowtan, 2004 ▸) and refined with *REFMAC*5 (Vagin *et al.*, 2004 ▸), which is a part of the *CCP*4 suite (Agirre *et al.*, 2023 ▸). Given the high resolution of 1.3 Å, anisotropic *B* factors were used in the final stages of the refinement. Data-collection and refinement statistics are summarized in Tables 3 ▸ and 4 ▸.

### 2.4. Differential scanning fluorimetry (DSF) measurements

The human or murine TRIM21 PRYSPRY domain at a concentration of 4 µ*M* was screened in a 384-well plate format against the LOPAC library at a concentration of 100 µ*M*. The compounds were mixed with protein using an Echo acoustic liquid handler. SYPRO Orange (5000×, Invitrogen) was added as a fluorescence probe at a volume of 1 µl per millilitre. Temperature-dependent protein-unfolding profiles were then measured using the *QuantStudio*5 real-time PCR system (Thermo Fisher), with the excitation and emission filters set to 465 and 590 nm, respectively. The temperature was increased at a rate of 3 K min^−1^. Data points were analysed using the internal *Protein Thermal Shift Software* (version 1.4, Thermo Fisher), applying the Boltzmann equation (*F* = *F*_min_ + (*F*_max_ − *F*_min_)/{1 + exp[(*T*_m_ − *T*)/*s*]}, where *F* is fluorescence and *T* is temperature) to determine the inflection point of the unfolding transition curve.

### 2.5. ITC measurements

ITC measurements were as executed as described previously (Krämer *et al.*, 2022 ▸). Briefly, human and murine TRIM21 were diluted with their respective SEC buffers to the desired concentration (final concentration 15–30 µ*M*) and loaded into the cell. Suramin was diluted from a 50 m*M* DMSO stock in the same buffer (final DMSO concentration 0.3–0.6%) and filled into the syringe. The DMSO concentration in the protein solution was adjusted accordingly. ITC measurements were performed using an Affinity ITC (TA Instruments) at a temperature of 293 K and a stirring rate of 75 rev min^−1^. The suramin solution was titrated into the protein solution (172 µl cell volume) with 2.5 µl per injection, except for the first injection, which was 1 µl. The time between each injection was set to 200 s. The baseline was corrected by performing a control experiment in which the suramin buffer solution was titrated into buffer without protein. Each ITC measurement was repeated at least three times. The results were analysed using the internal *NanoAnalyze* software (TA Instruments) using the single, reversible binding-site model. The curve was visualized with *GraphPad Prism* (https://www.graphpad.com).

## 3. Results

### 3.1. Screening of the LOPAC library and follow-up validation with ITC

**Figure 2:**
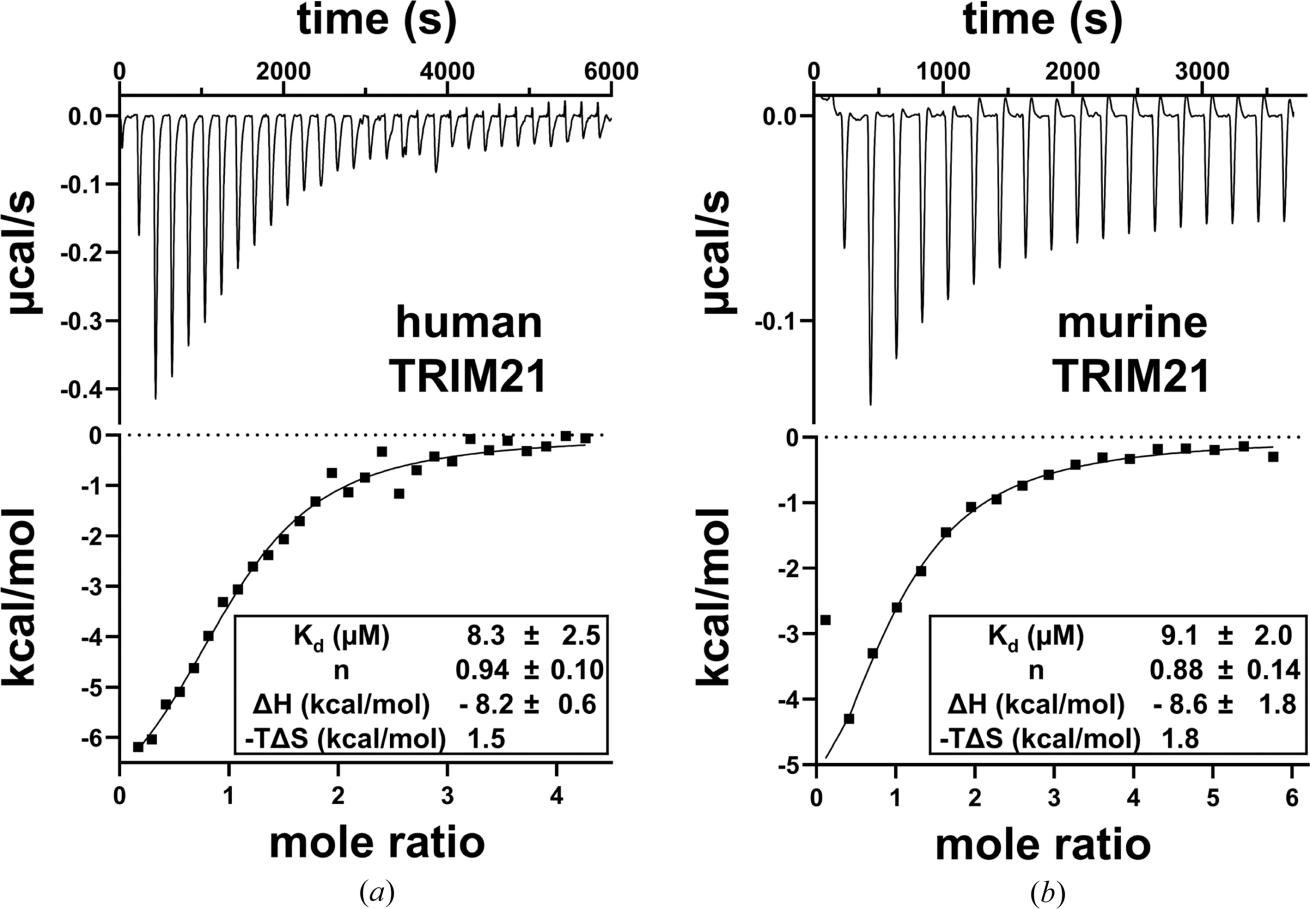
ITC curves of suramin binding to human (*a*) and murine (*b*) TRIM21 PRYSPRY domains. Titration heats of each injection (upper panel) and normalized binding heats (lower panel) are shown. The parameters of a nonlinear least-squares fit of the binding isotherm to a single, reversible binding-site model are shown in the image for each titration.

We screened the LOPAC library containing 1280 compounds at a concentration of 100 µ*M* against the PRYSPRY domain of human TRIM21 using a thermal shift assay, setting a melting-curve shift cutoff of 0.5 K. This screening approach identified AMG9810 (0.8 K), R59949 (0.5 K), SANT-2 (0.8 K) and suramin (2.1 K) as potential hits (DSF curves and ligand structures are shown in Supplementary Fig. S1). Follow-up ITC experiments confirmed the binding of suramin to human TRIM21 with an affinity (*K*_d_) of approximately 8 µ*M* (Fig. 2*a* ▸). However, no significant binding was observed for the other compounds using ITC.

In parallel, we attempted to obtain co-crystal structures for all identified compounds. Initial crystallization trials of human TRIM21 failed and therefore we used mouse TRIM21 as a surrogate due to its high sequence identity (75%) with human TRIM21 and its previously determined crystallization conditions (PDB entry 2vok; Keeble *et al.*, 2008 ▸). Co-crystallization attempts using the published conditions yielded crystals for all compounds except suramin. However, consistent with our ITC results, we observed no additional electron density accounting for the binding of AMG9810, R59949 or SANT-2. Notably, soaking apo crystals with suramin completely dissolved the crystals. This could indicate that suramin binds to a site that interferes with crystal contacts, among other possible reasons such as pH changes, ionic strength, increased DMSO concentration *etc*. We therefore successfully screened for new crystallization conditions using a pre-incubated protein with suramin and determined a high-resolution (1.3 Å) structure of the murine TRIM21–suramin complex, which revealed clear electron density for the ligand.

### 3.2. Crystal structure of the mouse TRIM21–suramin complex

**Figure 3:**
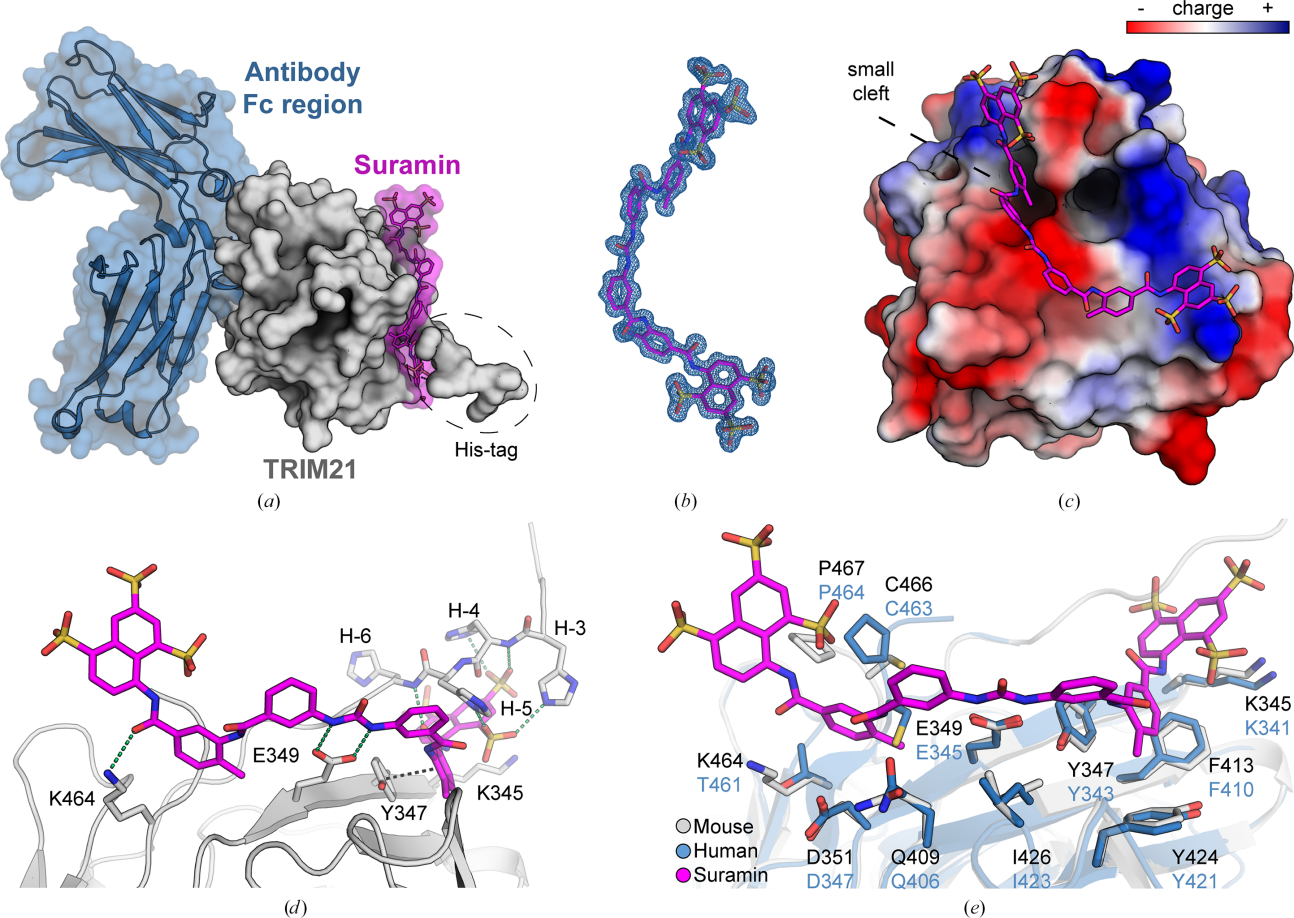
Crystal structure of the TRIM21–suramin complex. (*a*) Surface representation of mouse TRIM21 (grey) complexed with suramin (pink). For orientation, the binding site of the antibody Fc region (blue) is shown based on PDB entry 3zo0. The position of the N-terminal His-tag is indicated. (*b*) 2*F*_o_ − *F*_c_ electron-density map for suramin, contoured at 1σ (blue mesh). (*c*) Electrostatic surface-charge representation of TRIM21. The His-tag has been removed in this panel for a clearer view of the ligand and its artificial nature. Even without the tag this area remains positively charged. (*d*) Suramin binding to TRIM21. Hydrogen bonds and ionic interactions between positively charged histidine residues and negatively charged sulfonate groups are shown as green dashed lines and π interactions are shown as black dashed lines. Noncanonical residues from the His-tag are prefixed with a minus sign. (*e*) Comparison of residues within 5 Å of suramin between mouse (grey) and human (blue) TRIM21. The tag residues are removed in this panel.

The large molecule appears to be primarily surface-attached, mediated by a mixture of nonpolar, hydrogen-bond and electrostatic interactions between the negatively charged sulfonate groups and positively charged regions on the protein surface (Fig. 3 ▸*c*, Supplementary Fig. S2). Notably, one of the polysulfonated naphthyl moieties pointed away from the protein, with no direct interactions involving the sulfonate groups, whereas the same moiety on the other end interacted with the N-terminal His_6_-tag (Figs. 3 ▸*a* and 3 ▸*d*). However, despite these interactions with the His_6_-tag, the dissociation constant (*K*_d_) for the mouse protein (∼9 µ*M*; Fig. 2*b* ▸) was within the error range of the *K*_d_ measured for human TRIM21 (∼8 µ*M*) without the His_6_-tag. This observation may be explained by the fact that the protein surface in this area remains highly positively charged even without the tag (Fig. 3 ▸*c*). Key polar interactions involving side chains were observed between the central urea moiety of suramin and Glu349 and between Lys464 and one of the amide oxygens (Fig. 3 ▸*c*).

Interestingly, TRIM21 residues located within 5 Å of the suramin binding site are highly conserved between the human and mouse orthologues, even though the suramin binding site is located far from the substrate binding site. The only species-specific difference within this binding site is the substitution of lysine (Lys464) in the mouse protein with threonine in the human protein (Fig. 3 ▸*e*). The conservation of the binding site is highlighted by the similar affinities for suramin determined by ITC for both orthologues. According to these results, the binding of suramin is mainly enthalpy-driven, which is consistent with the observed polar, van der Waals and electrostatic interactions contributing to a favourable polar interactions and large negative binding enthalpies. Overall, the compound binding induced minimal structural changes, with only slight adjustments of side chains in the surrounding residues compared with the apo structure. A notable exception is the outward movement of the C-terminal region (Cys466 and Pro467) to form a pocket that was completely occupied by a tolyl moiety of suramin. These subtle conformational changes are visualized in Supplementary Video S1.

## 4. Discussion

The suramin–TRIM21 PRYSPRY complex structure revealed a unique binding site, which to the best of our knowledge has not previously been described as an interaction site and its functional relevance therefore remains unknown. A long-term objective could involve the design of degradation molecules, such as PROTACs or molecular glues, based on the observed binding site and interactions. A potential advantage of degraders targeting this site, compared with those targeting the antibody binding site, is that they would likely avoid interfering with substrate recruitment and the physiological E3 ligase function of TRIM21. However, optimizing the pharmacological properties of a compound such as suramin, addressing major issues such as toxicity, side effects and low binding affinity, represents a major challenge. Nonetheless, we believe that this compound holds substantial potential for optimization. For instance, the negative charge of the sulfonate groups restricts cell permeability, and several polar and charged moieties observed in the crystal structure are surface-exposed, suggesting the possibility of substituting them with less charged groups such as sulfonamides or sulfonyl groups, which would likely increase cell permeability. Furthermore, the high molecular weight of suramin presents a barrier, and a reduction in size could enhance its drug-like properties (Lipinski *et al.*, 2001 ▸). Its symmetrical structure suggests that the ligand could be reduced to half its size with minimal modifications, providing a foundation for further exploration and validation. Another promising strategy might focus on covalently targeting the cysteine inside the small cavity formed by the outward movement of the C-terminus. This approach could involve a smaller version of the ligand equipped with a covalent warhead, such as an acrylamide, as commonly used in the development of covalent inhibitors (Gehringer & Laufer, 2019 ▸).

## 5. Related literature

The following reference is cited in the supporting information for this article: Laskowski & Swindells (2011 ▸).

## Supplementary Material

PDB reference: TRIM21 PRYSPRY domain bound to suramin, 9gte

Supplementary Figures. DOI: 10.1107/S2053230X25000913/nq5001sup1.pdf

Video S1. Suramin binding to TRIM21: conformational changes upon suramin binding. DOI: 10.1107/S2053230X25000913/nq5001sup2.mp4

## Figures and Tables

**Table 1:** Macromolecule-production information for mouse and human TRIM21 PRYSPRY domains

Source organism	*Mus musculus*	*Homo sapiens*
Expression vector	pET-3d (N-terminal His_6_-tag)	pET His_6_ SUMO LIC
Expression host	*Escherichia coli*	*Escherichia coli*
Complete amino-acid sequence of the construct produced	MHHHHHHMVHITLDRNTANSWLIISKDRRQVRMGDTHQNVSDNKERFSNYPMVLGAQRFSSGKMYWEVDVTQKEAWDLGVCRDSVQRKGQFSLSPENGFWTIWLWQDSYEAGTSPQTTLHIQVPPCQIGIFVDYEAGVVSFYNITDHGSLIYTFSECVFAGPLRPFFNVGFNYSGGNAAPLKLCPLKM	**MCSSHHHHHHGSGSGSDQEAKPSTEDLGDKKEGEYIKLKVIGQDSSEIHFKVKMTTHLKKLKESYCQRQGVPMNSLRFLFEGQRIADNHTPKELGMEEEDVIEVYQEQTGG**VHITLDPDTANPWLILSEDRRQVRLGDTQQSIPGNEERFDSYPMVLGAQHFHSGKHYWEVDVTGKEAWDLGVCRDSVRRKGHFLLSSKSGFWTIWLWNKQKYEAGTYPQTPLHLQVPPCQVGIFLDYEAGMVSFYNITDHGSLIYSFSECAFTGPLRPFFSPGFNDGGKNTAPLTLCPL†

† Before cleavage. Residues in bold belong to the SUMO tag.

**Table 2:** Crystallization

Method	Vapour diffusion, sitting drop
Plate type	3-lens 96-well plate (SWISSCI)
Temperature (K)	293
Protein concentration (mg ml^−1^)	10
Buffer composition of protein solution	25 m*M* Tris–HCl pH 7.8, 150 m*M* NaCl, 0.5 m*M* TCEP
Composition of reservoir solution	20% PEG 6000, 0.2 *M* ammonium chloride, 10% ethylene glycol
Volume and ratio of drop	200 nl (1:1 ratio of protein:reservoir)
Volume of reservoir (µl)	20

**Table 3:** Data collection and processing Values in parentheses are for the outer shell.

Diffraction source	I03, Diamond Light Source
Wavelength (Å)	0.97625
Temperature (K)	100
Detector	EIGER2 XE 16M
Crystal-to-detector distance (mm)	201.016
Rotation range per image (°)	0.1
Total rotation range (°)	360
Exposure time per image (s)	0.0055
Space group	*P*2_1_2_1_2_1_
*a*, *b*, *c* (Å)	42.59, 56.02, 69.00
α, β, γ (°)	90, 90, 90
Mosaicity (°)	0.06
Resolution range (Å)	43.49–1.30 (1.32–1.30)
Total No. of reflections	939012 (15113)
No. of unique reflections	41260 (1896)
Completeness (%)	99.7 (94.8)
Multiplicity	22.8 (8.0)
〈*I*/σ(*I*)〉	22.1 (3.6)
*R*_meas._	0.092 (0.582)
Overall *B* factor from Wilson plot (Å^2^)	9.9

**Table 4:** Structure solution and refinement Values in parentheses are for the outer shell.

Resolution range (Å)	43.49–1.30 (1.32–1.30)
Completeness (%)	99.7 (94.8)
No. of reflections, working set	39175 (2766)
No. of reflections, test set	2020 (132)
Final *R*_cryst_	0.157 (0.214)
Final *R*_free_	0.177 (0.220)
No. of protein atoms	1535
No. of ligand atoms	86
No. of solvent atoms	24
No. of water atom	151
Total No. of atoms	1796
R.m.s. deviations	
Bond lengths (Å)	0.008
Angles (°)	1.428
Average *B* factors (Å^2^)	
Protein	10
Solvent	21.5
Ligand	11
Ramachandran plot	
Most favoured (%)	98
Allowed (%)	2
