# Supplementary material for: LOPAC library screening identifies suramin as a TRIM21 binder with a unique binding mode revealed by crystal structure
Source: Acta Crystallogr F Struct Biol Commun. 2025 Feb 16;81(Pt 3):101–7. doi: 10.1107/S2053230X25000913 (PMC11866408; doi:10.1107/S2053230X25000913)
Supplement: Supplementary file 1 [file f-81-00101-sup1.pdf]

STRUCTURAL BIOLOGY
COMMUNICATIONS

Volume 81 (2025)

Supporting information for article:

LOPAC library screening identifies suramin as a TRIM21 binder with a unique binding mode revealed by crystal structure

Yeojin Kim, Stefan Knapp and Andreas Krämer

S1. DSF data

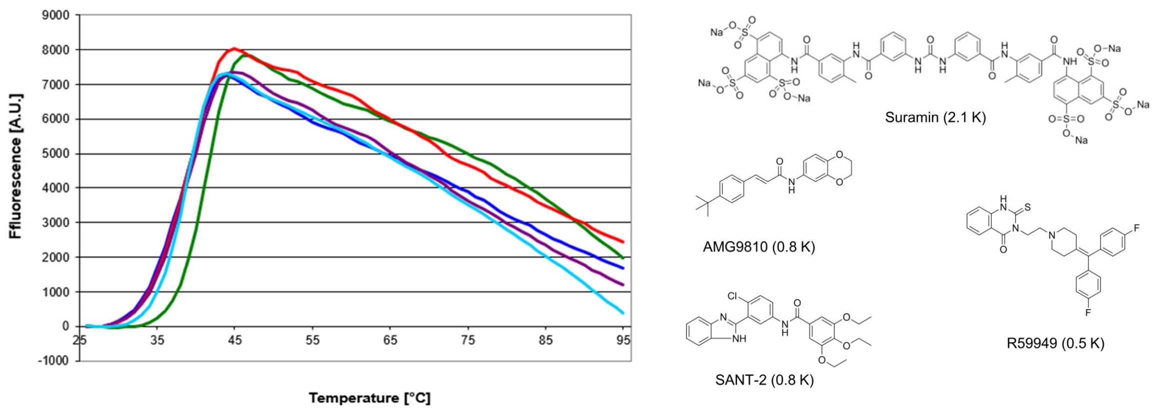

Figure S1 Melting curves of TRIM21 with different ligands. Dark blue is TRIM21 without inhibitor. Green is suramin, red is AMG9810, purple SANT-2 and light blue is R59949.Chemical structures are given on the right including thermal shift in Kelvin.

S2. Binding analyses with LigPlot+

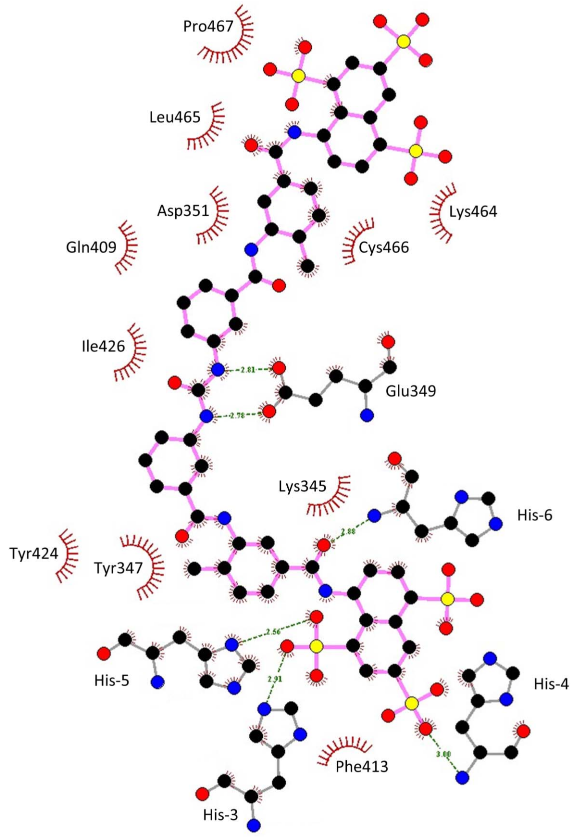

Figure S2 Ligand environment visualized and analysed with LigPlot+(Laskowski & Swindells, 2011). Polar interactions are indicated by green dashed lines.

S3. Multimedia Content

Video S1 Suramin binding to TRIM21: Conformational changes upon suramin binding

References

Laskowski, R. A. & Swindells, M. B. (2011). *J Chem Inf Model* **51**, 2778-2786.
